# ASSESSMENT OF COMPARATIVE EFFECTIVENESS RESEARCH GAPS TO PROMOTE AGING IN PLACE: A STAKEHOLDER-DRIVEN APPROACH

**DOI:** 10.1093/geroni/igz038.2237

**Published:** 2019-11-08

**Authors:** Gyasi Moscou-Jackson, Sindhura Gummi, Steven B Clauser, Neeraj K Arora

**Affiliations:** PCORI, District of Columbia, United States

## Abstract

A priority for many older adults is to remain in their homes and communities. This paper describes the Patient-Centered Outcomes Research Institute’s (PCORI) review of our investment in comparative effectiveness research (CER) that will advance the field of “aging in place.” To inform our systematic evaluation, we first engaged our multi-stakeholder advisory panel of patients/caregivers, clinicians, health systems leaders, and payers to develop and refine a conceptual framework for research focused on aging in place among older adults. Key themes from stakeholders were: aging in place interventions should be patient-centered and align with the patients’ needs and goals, studies should include individuals 55+ years who may be at risk for institutionalization, relevant interventions go beyond environmental modifications and include social support, healthcare (inclusive of palliative care), and personal care services. In addition, aging in place services should focus more on informal caregiver interventions and outcomes since informal caregivers play a major role in enabling older adults to age in place. We identified 14 PCORI-funded CER projects that will provide evidence on the most effective interventions to promote aging in place among older adults; the total investment is $113.4 million dollars. The portfolio is addressing decisional dilemmas faced by multiple stakeholders on a variety of topics including falls prevention, home-based palliative care, and community-based care models; however, several critical CER evidence gaps remain that need to be addressed in future funding investments and will be discussed during the presentation.

